# The Challenges of Conducting Clinical Research on Neglected Tropical Diseases in Remote Endemic Areas in Sudan

**DOI:** 10.1371/journal.pntd.0004736

**Published:** 2016-11-03

**Authors:** Sayda El-Safi, François Chappuis, Marleen Boelaert

**Affiliations:** 1 University of Khartoum, Khartoum, Sudan; 2 Division of Tropical and Humanitarian Medicine, Geneva University Hospitals, Geneva, Switzerland; 3 Department of Public Health, Institute of Tropical Medicine, Antwerp, Belgium; International Centre of Insect Physiology and Ecology, KENYA

## Introduction

Most neglected tropical diseases (NTDs) occur in remote areas of low- and middle-income countries, where health systems are often poorly developed. Therefore, these neglected patients generally lack access to quality preventive, diagnostic, and therapeutic care [1]. The difficulty to access NTD-endemic areas, challenging logistics, and the lack of skilled human resources in these areas are also major obstacles to conduct clinical research. We had to face these when conducting a good clinical practice/good clinical laboratory practice (GCP/GCLP)-compliant clinical study in eastern Sudan, a well-known endemic area for visceral leishmaniasis and other NTDs. We share here the challenges related to study preparation and implementation of this GCP/GCLP study that investigated the causes of persistent fever in a rural hospital located in Gedaref State in eastern Sudan. We think this type of paper may be of interest for researchers planning to conduct a clinical trial in a resource-limited setting as well as for funders of such research.

## Study concept

The introduction of rapid diagnostic tests (RDTs) for malaria in clinical practice in the tropics has been a game changer in the management of febrile illness, revealing the frequency of nonmalarial fevers and the long list of differential diagnosis [2,3]. Unfortunately, the limited laboratory capacity in these resource-constrained settings does not give much guidance to the clinicians who tend to prescribe a cocktail of drugs—including antibiotics—on an empirical basis. [1,4]. Five years ago, a team from the University of Khartoum, Sudan, joined the European research network on better diagnosis for neglected infectious diseases (NIDIAG) to investigate the differential diagnosis of the persistent fever syndrome, with the aim to develop evidence-based clinical guidance (www.nidiag.org). The study design was that of a descriptive, prospective clinical study to reach a final, confirmed diagnosis in the largest possible number of febrile patients (fever ≥7 days) with the support of national and international reference laboratories. Hence, the study required the shipment of a large number of samples within-country and abroad (S1 Fig). The knowledge on specific pathogens causing this persistent febrile syndrome would in a second stage be used to elaborate diagnostic guidance for the syndrome. We aimed to conduct this clinical study in compliance with the GCP/GCLP standards [5].

## Study preparation

### Protocol development

Protocol development took much longer than expected because of the intrinsic complexity of harmonising the study protocol for field sites situated in four countries (Sudan, the Democratic Republic of the Congo, Nepal, and Cambodia). More than a year elapsed between the circulation of the first draft protocol (November 2011) and the approval of version 1.0 (January 2013) (registered in ClinicalTrials.gov with identifier NCT01766830).

### Preparing for GCP/GCLP standards

For each procedure in the study, be it patient recruitment, physical examination, or diagnostic test, a standard operating procedure (SOP) was drafted [5]. In total, more than 40 SOPs were developed, reviewed, and approved during quarter 3 and quarter 4 of 2012. These SOPs are provided as supplemental material to the paper by Alirol et al. in this *PLOS Neglected Tropical Diseases* issue [6]. The preparation of the study sites (e.g., recruitment and training of research staff [S1 Table], including GCP/GCLP training, initiation monitoring visit, ordering of equipment and consumables) took place between quarter 3 of 2012 and quarter 1 of 2013. RDTs for malaria, leptospirosis, typhoid fever, and kala-azar were ordered and sent with temperature monitors from the manufacturers to the four countries during quarter 3 of 2012. The United States embargo imposed on Sudan hampered the import of research materials from US companies.

### Ethical clearance

Seven months passed between the first submission of the study protocol to an ethical committee in Europe and the clearance in all participating countries. We submitted the study protocol first to the Ethical Committee of the sponsor (University of Antwerp, Belgium) on 23 May 2012 and got approval on 25 June 2012. The National Ethics Committee for Health Research, Cambodia (Submission: 27 May 2012; Approval: 24 August 2012), the Nepal Health Research Council (Submission: 29 May 2012; Approval: 3 July 2012), the Ethical Committee of the University of Khartoum, Sudan (Submission: 3 June 2012; Approval: 13 June 2012), the National Research Ethics Review Committee, Sudan (Submission: 5 September 2012; Approval:18 November 2012), and the Comité d’Ethique de l’ Ecole de Santé Publique de l’Université de Kinshasa, DRC (Submission: 4 December 2012; Approval 14 January 2013) followed. The minor changes requested by the national committees were country-specific and did not require resubmission of the common protocol.

In brief, this study, which had looked relatively simple at the outset, took us almost two years of preparation. Protocol preparation and ethical clearance was a lengthy process, and the time required to develop the case report form (CRF) and SOPs was much longer than expected. Over to Sudan now to examine the challenges encountered during study implementation.

## Study implementation

### Study site selection

**Fig 1:**
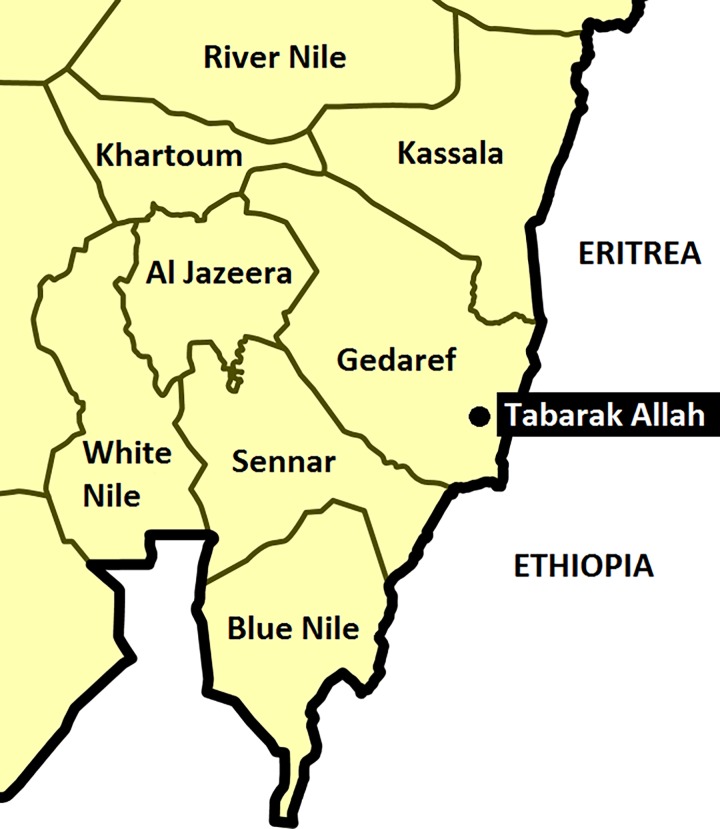
Study location. Tabarak Allah, Gedaref province, Sudan.

We established several criteria for the selection of the study site: (1) location in the most endemic area for visceral leishmaniasis and close to the referral hospital in Gedaref; (2) logistical capacity (24 hours electricity supply, cold chain, communication channels); and (3) reliable supply of medicines and food. With these criteria in mind, we selected Tabarak Allah (TBK) Rural Hospital in Gedaref State as the study site (Fig 1). This hospital is run by the Ministry of Health (MoH) and supported by Médecins sans Frontières (MSF) in a dedicated visceral leishmaniasis and nutrition programme. Our first challenge was to harmonise the work between the three partners (MoH, MSF, and NIDIAG) so as to avoid work duplication and disruption of clinical services by research activities. Firstly, we had to agree on the patient flow (S2 Fig) so that the patient would be initially registered at MoH, invited (if eligible) for enrolment in the NIDIAG study, and referred back to MSF for treatment if diagnosed with visceral leishmaniasis. Secondly, we had to resolve the differences between the NIDIAG and MSF (and to some extent, the NIDIAG and MoH) protocols in relation to visceral leishmaniasis case management. To that end, a memorandum of understanding was signed between the three partners.

### Logistics

We faced major logistical constraints. Tabarak Allah is located 200 km away from Gedaref town, and a large part of the road is black-cotton soil that gets impassable—even for 4x4 vehicles—during the rainy season. Gedaref town is located at about 600 km from Khartoum (Fig 1). Blood samples for biochemistry and other tests as well as inoculated culture media had to be carried weekly from Tabarak Allah to Khartoum, received on the same day at Suba University Hospital, and dispatched the next day to four central laboratories in the capital. The University of Khartoum made one car available for the study, but it was caught in an accident soon after the start of the study. Patients had to use public transport, but we reimbursed all fees related to patient transport.

### A false start

Patient enrolment started in Tabarak Allah Rural Hospital on 21 March 2013, and within about two weeks, 28 patients were recruited. However, on 8 April 2013 the recruitment had to be interrupted because a laboratory monitoring visit pointed to the poor quality of locally purchased materials and the lack of equipment and consumables that could not be purchased from the local market. This interruption was meant to last only two weeks—the time needed to import better quality laboratory products—after which recruitment would be resumed. Unfortunately, on the following day (9 April 2013), the study team had a severe car accident on their way from Gedaref to Khartoum, causing almost total damage to the car but fortunately only minor injuries to the team and the driver. The biological samples from six patients were lost in the accident. The study had to be interrupted for about five months instead of only two weeks. The study was restarted on 8 September 2013. From that day onwards, MSF kindly provided transport with hired tractors for the study team during the rainy season.

### Management issues

One of the biggest challenges we had to overcome was totally unexpected. The EU grant had to be transferred to the Sudanese principal investigator (PI) in several instalments. The first and second bank transfer went smoothly, but in July 2014, probably as a result of a recent court case in the US related to the embargo [7], several banks (including BNP Paribas Fortis and Deutsche Bank) refused to make the transfer of the EU funds from Belgium to Sudan, even if, strictly speaking, medical research does not fall under the embargo rules. The PI from the University of Khartoum had to keep the study going from her own personal resources—without any external financial support—for several months until we found an alternative channel for the transfer of funds.

### Human resources

One month after the restart of the study, the NIDIAG study physician in Tabarak Allah developed severe malaria and had to be brought back to Khartoum for treatment; after that episode he refused to return to Tabarak Allah. The site investigator worked single-handedly for about three months, as it was so difficult to recruit a second doctor because of the tough field conditions and mass emigration of young doctors—particularly to Saudi Arabia—for better job opportunities. In December 2013, the site investigator and one of the two NIDIAG site technicians developed an acute febrile illness and the study had to be interrupted again for two weeks.

### Patient recruitment

On average, 12 eligible patients with persistent fever were seen per day at Tabarak Allah Hospital, but only four participants could be enrolled per day because of the limited laboratory capacity. The NIDIAG study became popular in the area, as the news spread that investigations and other costs were taken care of by the project. Hence, more and more patients with fever syndrome came from further away. This led to a significantly increased workload: higher numbers of noneligible febrile patients and a higher rate of patients with noninfectious diseases (chronic diseases, gastritis, etc.) in the hospital. In addition, a lot of patients came back for scheduled as well as unscheduled follow-up visits (up to 15 patients per day).

### Patient follow-up

Access to the clinic facilities was maintained throughout the study period, so patient safety and well-being was not compromised at any point during the study. Patients were given follow-up cards and the doctor kept all the essential information (telephone number, village address, name of tribe chief) so as to be able to reach the patient easily in case a patient did not come for a scheduled follow-up visit. Active follow-up was not feasible because of limited human and logistic (i.e., car) resources on site. Only 64% (431) patients completed their one-month follow-up visit. In light of all the constraints, this seems a reasonable follow-up rate.

### Laboratory issues

A large amount of time and energy went into the selection, purchase, and supply of the required diagnostic tests. HIV kits were supplied by the Sudanese National AIDS Programme. First, we used SD Bioline HIV 1/2 as a screening test, in line with the Sudanese HIV testing algorithm, and we developed study SOPs and CRF accordingly. However, when the Sudanese AIDS Programme changed its algorithm, the study also switched to Determine HIV 1–2 and the study documents had to be changed. In addition, we were out of stock of Determine HIV 1–2 tests for about three weeks and had to store the samples for delayed testing. We faced several issues with the supply of the required RDTs. Because of delays with the start of patient recruitment, some lots of rK39 and rK28 RDT for visceral leishmaniasis were already expired when the study started on 21 March 2013 or would expire shortly thereafter. These problems were solved by ordering new tests (rK28) and thanks to donation of test kits (rK39) by the MoH and MSF. Moreover, there were several issues with the quality of imported RDTs that will be discussed elsewhere (Barbé et al. 2016, in this issue) [8]. In addition, because of the US embargo, it was not possible to import some index tests directly from the manufacturers.

There was no suitable equipment for biochemistry assays available on site in Tabarak Allah. The original plan was for the NIDIAG technician at Suba University Hospital in Khartoum to perform biochemistry assays under the supervision of a specialist. Accordingly, the clinical samples had to be transferred weekly to the Leishmania Research Laboratory (where all NIDIAG activities were carried out). Optimising the available biochemistry equipment (Photometer 4040 from Riele, Berlin, Germany) for the biochemistry procedures at this laboratory was problematic, so the actual testing was done retrospectively on frozen samples by technologists at the Chemical Pathology Department of Suba University Hospital.

Contamination of *Leishmania* cultures at field level was frequent and required retraining of laboratory technicians and changes in the preparation of culture media. In addition, direct agglutination testing for visceral leishmaniasis was at times delayed for some patients because of unavailability of the MSF laboratory technician.

Transport of samples under cold chain conditions between the field site and Khartoum was one of the biggest challenges. It was planned once a week on Saturdays, and samples had to arrive on the same day of dispatch. MSF transported the samples from Tabarak Allah to Gedaref town, where the samples were delivered at the MSF office. A driver belonging to the research team of the University of Khartoum picked up the sample the same day at the MSF office in Gedaref and transported them to Khartoum, a 600 km, ten-hour drive that was not without troubles and hazards (see above). The NIDIAG driver sometimes had to travel further to Doka or even to Tabarak Allah and often arrived in Khartoum around 3 a.m. In Khartoum, the samples had then to be dispatched to four different laboratories. Needless to say, the transport had to be run with the rigor of a military operation (see Box 1).

**Box 1. Logistics of Sample Dispatch and Communication of Lab Results**

- Every Friday: Lab team in Tabarak Allah (TBK) prepares the shipments of samples.
- Every Saturday: Transport from Khartoum to TBK of original result forms and transport from TBK to Khartoum of samples.
- Every Saturday night: Reception of samples from TBK at Suba University Hospital (SUH).
- Every Sunday morning: Laboratory manager picks up samples at SUH and dispatches samples to four central laboratories: Veterinary Research Institute (VRI) for *Brucella* work, Faculty of Medicine (FOM) for *Salmonella* work, Leishmaniasis Research Laboratory (LRL) for *Leishmania* culture, and the National Health Laboratory (NHL) for tuberculosis culture.
- Every Thursday: Laboratory manager picks up blood cultures from FOM; takes them to VRI for *Brucella* work; collects results from FOM, VRI, and NHL laboratories; photocopies all the results forms; and gives the original copies to the driver to take them to TBK the next day.

### Data entry and management

Data entry could not be carried out on site in Tabarak Allah because of lack of facilities, space, and trained personnel, so we organised it in Khartoum. Data management was supervised by the GCP/GCLP unit of the Institute of Tropical Medicine Antwerp. We organised two training workshops on GCP-compliant data management, and continuous support was given to the data managers in Khartoum by email, telephone, and/or internet calls. Phone and internet connections were sometimes poor and did not allow any communication between the two teams. Our ambitious plan was to complete the first data entry within one month by training enough data entry clerks. This urgency was motivated by the fact that we wanted to retain the site investigator to respond to data queries, as he was about to migrate out to a Gulf country. A first workshop was rapidly organised to train data entry clerks. We had to organise later a second workshop, as the initial number of clerks was not sufficient to complete data entry on time. The main difficulty we encountered though was the transfer of the CRFs from Tabarak Allah to Khartoum. CRFs had to be transported weekly in batches from TBK to FOM. The road conditions were so bad during the rainy season that we could not get a single CRF out of Tabarak Allah for up to one month, even with a 4x4 vehicle.

### GCP/GCLP training

A formal three-day GCP/GCLP training was conducted in Khartoum prior to the study start for the study staff. As it was difficult to retain staff in tough field conditions, several field staff members—including a doctor, four internal quality managers, and two technologists—resigned during the study and had to be replaced. The newly appointed staff did not have access to an extensive three-day training workshop and were trained on site. The newly recruited collaborators were advised to access the GCP/GCLP documents on the NIDIAG website. The external monitors played an important role in training the newly appointed staff by running a few teaching sessions on GCP/GCLP during their monitoring visits in December 2013 and March 2014. Last but not least, the organization of the external monitoring was challenging. One visit planned in September 2013 had to be postponed for security reasons because of some instability in the country at that time. Additionally, it was difficult to obtain the required travel permits for the international monitors within 24 hours of their arrival in Khartoum, which they needed in order to spend the maximum possible time in the field.

## Discussion

This paper relates the major hurdles one has to overcome when conducting clinical research on NTDs (Table 1). It is likely that our research group, mainly composed of researchers based in European institutions or reference academic institutions located in capitals or large cities of NTD-endemic countries, (somewhat) underestimated the field and regulatory constraints of running a GCP/GCLP-compliant diagnostic study in rural Sudan, and this explains the long preparation period. In particular, the requirements of clinical microbiology expertise at field level were underestimated during the planning phase and proposal development. The real magnitude of the challenge was later realised, when storage and transport of samples had to be concretely planned and reference laboratories able to perform the reference tests had to be identified. In Khartoum, samples had to be dispatched to four different central laboratories, not to mention the regulatory difficulties and huge paperwork required to ship biological samples to reference laboratories in Europe.

**Table 1:** NIDIAG study issues identified in Sudan and lessons learnt.

Issues in the study	Lessons learnt
**I. Human resources**	
- NIDIAG/MSF/MoH collaboration - Illnesses and resigning of study staff - Increasing work load due to popularity of study - Low expertise of staff in clinical microbiology at field level	- Plan enough full-time equivalents for essential study staff in the budget, including the staff required for the implementation of the GCP/GCLP package. - Make sure that a clinical research site in a remote area can rely on enough medical doctors to fill in as back up. - Make sure that staff from non-malarial endemic areas receive proper anti-malarial prophylaxis when posted in endemic areas. - Provide enough incentives to field workers - Invest in the training of the laboratory technicians.
**II. Study preparation**	
- Protocol development took much longer than expected - Preparing for GCP/GCLP took much longer than expected - Ethical clearance of the study protocol took longer than expected - Low expertise of staff in clinical microbiology	- Take into consideration the substantial time required for protocol development in the time frame of a multicentre study. The time may be shortened by developing the draft protocol in a short workshop in which all the country PIs participate, instead of doing it sequentially. - Limit the number of SOPs. The drafting of SOPs can be equally be done collectively in a workshop involving the GCP/GCLP experts and the country principle investigators. - Work with external monitors, but in conjunction with quality managers on site, who should get good GCP/GCLP training. The quality managers can then train and supervise study staff who newly join the project. - Submit early and in parallel (rather than sequentially) the study protocol to the various ethical review boards. - Purchase and dispatch laboratory materials and tests (e.g. RDTs), taking the study realistic starting date into account. Timing is critical as tests and reagents have a limited shelf life.
**III. Logistics**	
- Accessibility due to rural setting & seasonal rains - Referral of severely sick patients - Procurement of lab supplies and consumables - Transport of samples	- Select a study site that is accessible during the rainy season and as close as possible to the referral hospital. The study budget should provide for the purchase of an appropriate vehicle if necessary. - Increase the laboratory capacity at the field study site by establishing the maximum number of diagnostic tests on site (e.g. biochemistry, tuberculosis microscopy). This will be both cost- and time-saving considering the 'military operation' that is required for moving samples from remote areas to reference laboratories in the capital city. - Increase the laboratory capacity of one central reference laboratory in which all reference tests that cannot be done in the study site are centralised. This will reduce the number of personnel, efforts of coordination, time to dispatch samples and facilitate quality assurance procedures.
**IV. Study management**	
- Transfer of study funds - Organisation of external monitoring - Limited laboratory capacity - Limited enrolment capacity - Limited capacity for data entry and management	- In the specific case of Sudan, we advocate for the lifting of the US embargo for research purposes. - Carefully plan GCP/GCLP monitoring visits, including a pre-recruitment visit of both clinical and laboratory monitors to ensure that all is OK on site before the start of the study. - Bottlenecks for enrolment should be documented and remediated timely. In our case we should have arranged for dedicated laboratory space separate from the clinical services laboratory. - Plan data entry facilities and train clerks for data entry at field level. Start data entry as early as possible. Improve internet connections in the field. In settings with very good internet access, satellite-based database access at the site of patient recruitment may be possible so that data entry could occur at the site where the CRFs are generated and remote monitoring could to some extent replace on-site monitoring.

In addition, the multicentre design of the study, taking place in four very different countries and settings, prolonged the preparation phase because of the longer-than-expected time needed to develop a common study protocol and to obtain ethical clearances from six ethical review committees, not to mention the writing of over 40 SOPs, the purchase of equipment and consumables, and the GCP/GCLP training of local investigators. In such studies, both researchers and funders should ensure that sufficient preparation time and budget is allocated prior to the start of patient recruitment. As described above, our study had to be interrupted after two weeks because some locally acquired laboratory consumables were missing or were of insufficient quality. This could have been avoided if the GCLP monitor had visited the site earlier, during the preparation phase. One of the most important lessons we learnt as a study team was that a substantial budget has to be earmarked for the GCP/GCLP package in clinical studies. This includes the staff time required for preparation of study documents and SOPs, the financing of GCP/GCLP monitoring, and the training of study personnel.

**Fig 2:**
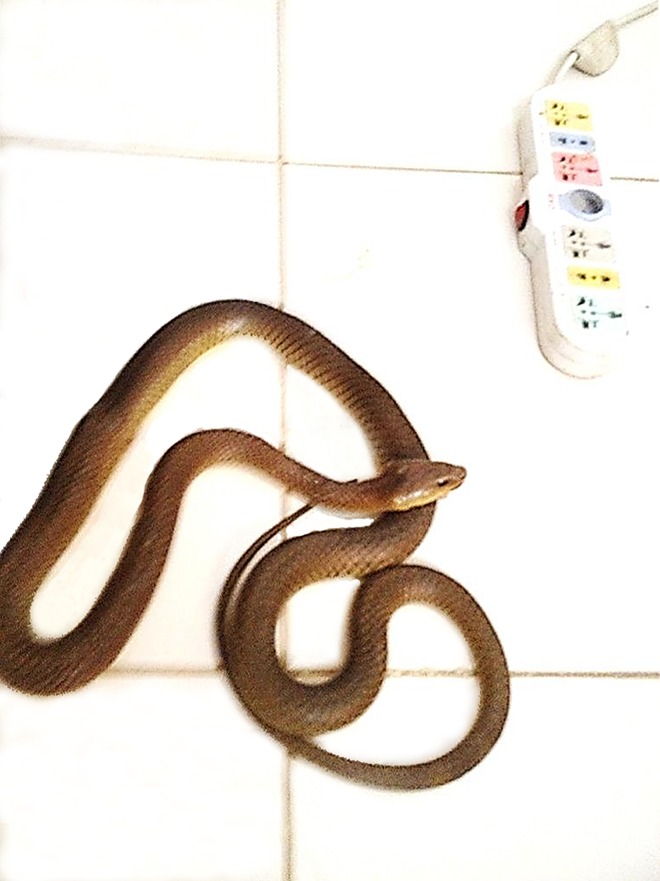
Unexpected visitor during a GCP/GCLP training workshop in Tabarak Allah Health Centre, Sudan.

The US embargo that is currently being imposed in Sudan added significantly to the complexity of our study, but with hindsight, the major bottleneck in this project was the lack of skilled human resources. In spite of the fact that the study doctors were generously paid in comparison to national standards, we experienced great difficulty in retaining our NIDIAG-trained doctors and in recruiting new ones. This may be partly related to the tough field conditions, including the risk of contracting severe infections or snake and/or scorpion bites (Fig 2). Sudan also faces a major brain drain of newly trained doctors, particularly towards Gulf countries.

In spite of all the delays encountered, the study site in Sudan was able to recruit the targeted number of participants within the planned time frame. We attribute this first and foremost to the fact that we had a highly motivated and dedicated team, which we could strengthen by adding a study doctor and laboratory technician. This study team worked seven days on seven, without interruption. An additional factor explaining the high recruitment rate was that the study site became highly popular in the region and attracted patients from a much larger catchment area, even from other states.

In conclusion, this study is considered by the Sudanese investigators as the toughest study they conducted since the start of their research career in 1986, though very rewarding. Against all odds, they achieved the required sample size within the planned time frame and obtained interesting results that will lead to better clinical care for patients at the primary health care level. The study gave them the stimulus to broaden their research scope and capacity to include other NTDs and to strengthen existing or establish new research collaborations. Most importantly, it brought European and Sudanese academic researchers together in the common quest to fulfil their mandate of social accountability.

## Supporting Information

S1 Fig Study specimen flowchart (DOCX)

S2 Fig Study flowchart for initial visit (DOCX)

S1 Table Capacity building of the Sudanese personnel for the NIDIAG study (DOCX)
