# Supplementary material for: The Challenges of Conducting Clinical Research on Neglected Tropical Diseases in Remote Endemic Areas in Sudan
Source: PLoS Negl Trop Dis. 2016 Nov 3;10(11):e0004736. doi: 10.1371/journal.pntd.0004736 (PMC5094669; doi:10.1371/journal.pntd.0004736)
Supplement: S1 Fig — (DOCX) [file pntd.0004736.s001.docx]

**STUDY SPECIMEN FLOWCHART – INITIAL VISIT SPECIMEN**
