# Supplementary material for: The Challenges of Conducting Clinical Research on Neglected Tropical Diseases in Remote Endemic Areas in Sudan
Source: PLoS Negl Trop Dis. 2016 Nov 3;10(11):e0004736. doi: 10.1371/journal.pntd.0004736 (PMC5094669; doi:10.1371/journal.pntd.0004736)
Supplement: S2 Fig — (DOCX) [file pntd.0004736.s002.docx]

**STUDY FLOWCHART FOR INITIAL VISIT**

Patient presenting with fever ≥1 week visits PHC & meets the medical registry personnel & referred to NIDIAG Team

Study investigator registers the patient in the patient identification list

Site investigator

checks for inclusion/exclusion

Criteria

Patient not included in the study

No

Yes

Provide the patient with the ICF or Assent

Patient refuses

Patient accepts

Continue with regular PHC plan/protocol of management

Patient sent to the lab for samples preparation with lab request form

1. Assign unique study number to the patient 2. Fill 2 forms of the ICF/ Assent & give one original copy to patient 3. Conduct a baseline assessment by filling the CRF

Lab technician fill in the study specimen log

Patient referred back to clinician for verifying working diagnosis & further management in collaboration with health provider

Transfer the patient to referral hospital for further investigations & management further inv. & management

Admission, daily visit till discharge decision made by health provider

Discharge, coordinate with health provider for arrangement of further FU visits

STANDARD FLOW CHART SYMBOLS WITH DESCRIPTIONS: (ACCORDING TO EACM & ISO RECOMMENDATIONS)

| **NO** | **SHAPE** | **DESCRIPTION** | **NO** | **SHAPE** | **DESCRIPTION** |
| --- | --- | --- | --- | --- | --- |
| 1 |  | PROCESS | 15 |  | CARD |
| 2 |  | ALTERNATE PROCESS | 16 |  | PUNCHED CARD |
| 3 |  | DECISION | 17 |  | SUMMING JUNCTION |
| 4 |  | DATA I/O | 18 |  | OR |
| 5 |  | PREDEFINED PROCESS | 19 |  | COLLATE |
| 6 |  | INTERNAL STORAGE | 20 |  | SORT |
| 7 |  | DOCUMENT | 21 |  | EXTRACT |
| 8 |  | MULTIPLE DOCUMENTS | 22 |  | MERGE |
| 9 |  | STARTER/ TERMINATOR | 23 |  | STORED DATA |
| 10 |  | PREPARATION | 24 |  | DELAY |
| 11 |  | MANUAL INPUT | 25 |  | SEQUENTIAL ACCESS STORAGE |
| 12 |  | MANUAL OPERATION | 26 |  | MAGNETIC DISK |
| 13 |  | CONNECTOR | 27 |  | DIRECT ACCESS STORAGE |
| 14 |  | OFF PAGE CONNECTOR | 28 |  | DISPLAY |

**Process / Operation Symbols**

| Symbol | Name (alias) | Description |
| --- | --- | --- |
| 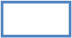 | Process | Show a Process or action step. This is the most common symbol in both process flowcharts and process maps. |
| 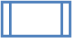 | Predefined Process (Subroutine) | A Predefined Process symbol is a marker for another process step or series of process flow steps that are formally defined elsewhere. This shape commonly depicts sub-processes (or subroutines in programming flowcharts). If the sub-process is considered "known" but not actually defined in a process procedure, work instruction, or some other process flowchart or documentation, then it is best not to use this symbol since it implies a formally defined process. |
| 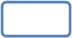 | Alternate Process | As the shape name suggests, this flowchart symbol is used when the process flow step is an alternate to the normal process step. Flow lines into an alternate process flow step are typically dashed. |
| 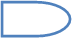 | Delay | The Delay flowchart symbol depicts any waiting period that is part of a process. Delay shapes are common in process mapping. |
| 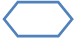 | Preparation | As the names states, any process step that is a Preparation process flow step, such as a set-up operation. |
| 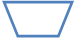 | Manual Operation | Manual Operations flowchart shapes show which process steps are not automated. In data processing flowcharts, this data flow shape indicates a looping operation along with a loop limit symbol (which is not supported by Microsoft Office, but a Manual Operation symbol rotated 180° will do the trick.) |

**Branching and Control of Flow Symbols**

| Symbol | Name (alias) | Description |
| --- | --- | --- |
| 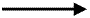 | Flow Line  (Arrow, Connector) | Flow line connectors show the direction that the process flows. |
| 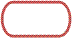 | Terminator (Terminal Point, Oval) | Terminators show the start and stop points in a process. When used as a Start symbol, terminators depict a*trigger action* that sets the process flow into motion. |
| 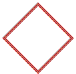 | Decision | Indicates a question or branch in the process flow. Typically, a Decision  flowchart shape is used when there are 2 options (Yes/No, No/No-Go, etc.) |
| 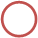 | Connector (Inspection) | **Flowchart**: In flowcharts, this symbol is typically small and is used as a Connector to show a jump from one point in the process flow to another. Connectors are usually labeled with capital letters (A, B, AA) to show matching jump points. They are handy for avoiding flow lines that cross other shapes and flow lines. They are also handy for jumping to and from a sub-processes defined in a separate area than the main flowchart. **Process Mapping**: In process maps, this symbol is full sized and shows an Inspection point in the process flow.  *[Just to confuse things further, some people will use a circle to indicate an operation and a square to indicate an inspection. That's why it's important to include a symbol key in the flowchart.]* |
| 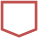 | Off-Page Connector | Off-Page Connector shows continuation of a process flowchart onto another page. When using them in conjunction with Connectors, it's best to differentiate the labels, e.g. use numbers for Off-Page Connectors and capital letters for Connectors. In actual practice, most flowcharts just use the Connect shape for both on-page and off-page references. |
| 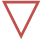 | Merge (Storage) | **Flowchart**: Shows the merging of multiple processes or information into one. **Process Mapping**: commonly indicates storage of raw materials. |
| 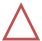 | Extract (Measurement) | **Flowchart:** Shows when a process splits into parallel paths. Also commonly indicates a Measurement, with a capital 'M' inside the symbol. **Process Mapping:** commonly indicates storage of finished goods. |
| 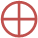 | Or | The logical Or symbol shows when a process diverges - usually for more than 2 branches. When using this symbol, it is important to label the out-going flow lines to indicate the criteria to follow each branch. |
| 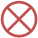 | Summing Junction | The logical Summing Junction flowchart shape is shows when multiple branches converge into a single process. The merge symbol is more common for this use, though. This symbol and the Or symbol are really more relevant in data processing flow diagrams than in process flowcharts. |

**Input and Output Symbols**

| Symbol | Name (alias) | Description |
| --- | --- | --- |
| 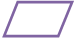 | Data (I/O) | The Data flowchart shape indicates inputs to and outputs from a process. As such, the shape is more often referred to as an I/O shape than a Data shape. |
| 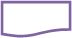 | Document | Pretty self explanatory - the Document flowchart symbol is for a process step that produces a document. |
| 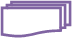 | Multi-Document | Same as Document, except, well, multiple documents. This shape is not as commonly used as the Document flowchart shape, even when multiple documents are implied. |
| 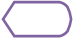 | Display | Indicates a process step where information is displayed to a person (e.g., PC user, machine operator). |
| 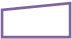 | Manual Input | Manual Input flowchart shapes show process steps where the operator/ user is prompted for information that must be manually input into a system. |
| 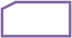 | Card | This is the companion to the punched tape flowchart shapes. This shape is seldom used. |
| 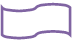 | Punched Tape | If you're very good at stretching all the life out of a machine, you may still have use for the Punched Tape symbol - used for input into old computers and CNC machines. |

**File and Information Storage Symbols**

| Symbol | Name (alias) | Description |
| --- | --- | --- |
| 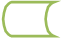 | Stored Data | A general Data Storage flowchart shape used for any process step that stores data (as opposed to the more specific shapes to follow next in this table). |
| 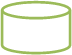 | Magnetic Disk (Database) | The most universally recognizable symbol for a data storage location, this flowchart shape depicts a database. |
| 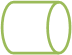 | Direct Access Storage | Direct Access Storage is a fancy way of saying Hard Drive. |
| 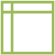 | Internal Storage | Used in programming flowcharts to mean information stored in memory, as opposed to on a file. |
| 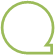 | Sequential Access Storage (Magnetic Tape) | Although it looks like a 'Q', the symbol is supposed to look like a reel of tape. |

**Data Processing Symbols**

| Symbol | Name (alias) | Description |
| --- | --- | --- |
| 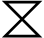 | Collate | The Collate flowchart shape indicates a process step that requires organizing data, information, or materials according into a standard format or arrangement. |
| 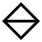 | Sort | Indicates the sorting of data, information, materials into some pre-defined order. |

Caveat: The descriptions given above are written from the perspective of someone using them to create**process flow charts**, whereas many of the flowchart symbols actually have their roots in the data processing diagrams and programming flow charts. So, not all the flowcharting shapes shown below may be relevant to your needs.

The flowchart symbol names in parentheses are common alternates for a given shape. Sometimes a flowchart shape can have more than one meaning, as with the Circle shape, which depicts a jump node in flow charts and inspection point in business process mapping. Other times, there are just multiple names for the same flow chart symbol - such as Terminator and Terminal Point.
