# Supplementary material for: The Challenges of Conducting Clinical Research on Neglected Tropical Diseases in Remote Endemic Areas in Sudan
Source: PLoS Negl Trop Dis. 2016 Nov 3;10(11):e0004736. doi: 10.1371/journal.pntd.0004736 (PMC5094669; doi:10.1371/journal.pntd.0004736)
Supplement: S1 Table — (DOCX) [file pntd.0004736.s003.docx]

Supporting information table: Capacity building of the Sudanese personnel for the NIDIAG study

| Category of personnel | Number trained | Trained on | | | | | | Location of training | | Trained by | |
| --- | --- | --- | --- | --- | --- | --- | --- | --- | --- | --- | --- |
|  |  | GCP | GCLP | Study protocol | Informed consent | CRF | SOP | Study site | Khartoum | Sudanese trainer | Foreign Trainer |
| Physicians | 11 | 11 | 0 | 6 | 4 | 4 | 4 | 6 | 5 | 0 | 11 |
| Laboratory technicians | 11 | 0 | 11 | 5 | 0 | 3 | 11 | 3 | 8 | 0 | 11 |
| Data entry clerks | 10 | 0 | 0 | 10 | 0 | 10 | 10 | 0 | 10 | 10 | 0 |

CRF: case report form; GCLP: good clinical laboratory practices; GCP: good clinical practices; SOP: standard operating procedure
